# AI-Driven Digital Twins for Manufacturing: A Review Across Hierarchical Manufacturing System Levels

**DOI:** 10.3390/s26010124

**Published:** 2025-12-24

**Authors:** Phat Nguyen, Minjung Kim, Elaina Nichols, Hwan-Sik Yoon

**Affiliations:** Department of Mechanical Engineering, The University of Alabama, Tuscaloosa, AL 35487, USA; pvnguyen@crimson.ua.edu (P.N.); mkim72@crimson.ua.edu (M.K.); esnichols@crimson.ua.edu (E.N.)

**Keywords:** digital twin, artificial intelligence, smart manufacturing, predictive maintenance, process optimization, quality control

## Abstract

Digital Twins (DTs) integrated with Artificial Intelligence (AI) are emerging as transformative tools in smart manufacturing. By bridging the physical and virtual domains, DTs enable real-time monitoring, predictive analytics, and autonomous decision-making. Originally conceived as advanced simulation models, DTs have evolved significantly with the incorporation of AI, which enhances their ability to acquire process knowledge, optimize scheduling, and autonomously control system variables. This evolution transforms DTs from passive representations into prescriptive, self-optimizing systems. AI-driven DTs support a wide range of applications, including predictive maintenance, process optimization, quality control, and dynamic scheduling, using techniques such as deep reinforcement learning and convolutional neural networks. These capabilities have been successfully deployed across industrial domains such as CNC machining, robotics, and industrial printing, yielding substantial improvements in efficiency, reliability, and responsiveness. Despite these advancements, the full realization of intelligent DTs relies heavily on the availability of high-fidelity, real-time data and a seamless alignment between physical systems and their digital counterparts. This literature survey provides a state-of-the-art review of AI-driven DTs in manufacturing, highlighting their key applications, challenges, and emerging research directions that will shape the future of intelligent and adaptive manufacturing systems. To present a structured perspective on the evolution and scalability of AI-driven DTs, the application case studies are organized according to four integration levels—machine, cell, shop floor, and enterprise—highlighting how these technologies scale from individual assets to fully interconnected manufacturing ecosystems.

## 1. Introduction

Smart manufacturing is a transformative approach to industrial production, characterized by the integration of advanced technologies such as the Internet of Things (IoT) [1,2], cyber–physical systems, and real-time data analytics. Smart manufacturing supports highly flexible, efficient, and adaptive manufacturing systems that can rapidly respond to various changes in product demand, production conditions, or design specifications. With the current digitalization trend across industries, a large volume of data is now available to manufacturers. However, much of this information remains underutilized and locked away in “dark data” silos [3,4]. As manufacturing systems become increasingly complex, the need for intelligent, data-driven tools that can model, monitor, and optimally control manufacturing operations in real time is becoming ever more important.

One of the key components supporting this technological transformation is the Digital Twin (DT) [5]. First introduced by Grieves in 2002 [6], DTs serve as virtual replicas of physical systems or processes, continuously updated through data exchange with their real-world counterparts. This combination of physical and virtual spaces provides manufacturers with more clear and detailed information of system operations [7]. A DT typically consists of a physical system, its digital model, and bi-directional data exchange between the two. Key features such as high-fidelity reflection, constant interaction, and self-improvement through data exchange allow DTs to provide dynamic, real-time insights into the physical system and its operation. DTs can replicate the behavior of individual machines or entire production lines, enabling manufacturers to run simulations, predict system performances, and make informed decisions without interrupting ongoing operations. In practice, DTs are applied to predictive maintenance, process optimization, quality control, and lifecycle management at various integration levels.

Recent advancements in Artificial Intelligence (AI) have further expanded the capabilities of DTs by helping them learn complex system behaviors, adapt to changing conditions, and make data-driven predictions. When integrated with DT frameworks, AI algorithms are able to process large-scale sensor and operational data to detect system anomalies, optimize control strategies, and even automate decision-making processes. For instance, reinforcement learning can be used to determine optimal process parameters in real time, while Deep learning (DL) models can enhance the accuracy of virtual sensors within the DT. As a result, AI-integrated DTs not only improve performance and reliability but also enable innovation in autonomous and self-optimizing manufacturing systems.

Although several comprehensive reviews have examined the integration of AI and DTs in the context of smart manufacturing, the rapid pace of technological advancement continues to generate new methodologies, frameworks, and application domains. In particular, emerging trends such as hybrid models, federated learning, interpretable AI, and scalable DT architectures are continuously reshaping the landscape. As a result, there is a need for an up-to-date synthesis that not only captures recent developments but also provides critical insights into ongoing challenges and future research directions. This review paper seeks to address that need by building upon existing literature and offering a current perspective on the convergence of AI and DT technologies for next-generation smart manufacturing systems. Specific contributions of this paper include foundational concepts and the evolution of DTs, AI methodologies enhancing DT capabilities, practical case studies organized across machine, cell, shop floor, and enterprise levels to demonstrate tangible DT impacts, as well as the identification of critical research gaps and future research opportunities.

The remaining sections are organized as follows. The background and previous review studies are presented in Section 2, and the current integral role of AI in the continuing advancement of DT technology is discussed in Section 3. Next, common DT applications in manufacturing with real world examples from case studies organized by machine, cell, shop floor, and enterprise levels are highlighted in Section 4. Then, current challenges and limitations in the expansion of the DT domain are discussed in Section 5. Future research opportunities are presented in Section 6, and finally the literature survey is concluded in Section 7.

## 2. Background and Previous Review Studies

This section introduces the core concept of DT for this review, summarizes previous review studies related to DT applications in manufacturing, and explains the motivation for adopting a manufacturing hierarchy-level perspective and functional categories.

### 2.1. Core Concept of Digital Twins

DTs are dynamic virtual representations of physical systems, evolving from simple digital replicas to sophisticated platforms that enable system-level interaction and decision-making. The concept of DTs is often traced to Grieves’s presentation on product lifecycle management [6]. According to Kritzinger et al. [8], DTs are distinguished from related concepts such as digital models and digital shadows with emphasis placed on the bidirectional exchange of data and influence between physical and virtual entities.

**Figure 1:**
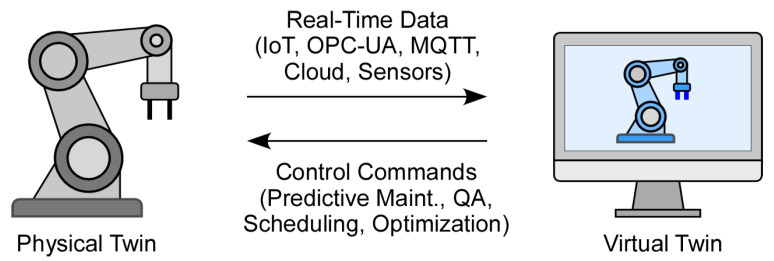
Basic architecture of Digital Twin systems with three components.

According to Grieves, a DT should span the entire lifecycle of its physical counterpart from pre-construction to post-disposal [6]. This lifecycle typically includes phases such as design, implementation, operation, and retirement. Most foundational DT architectures, regardless of domain, comprise three core components. As shown in Figure 1, these components are: (1) the physical twin, which includes the manufacturing asset and its operating environment; (2) the virtual twin, a continuously updated digital representation of the physical system; and (3) the bidirectional, synchronized data connections between the two, enabling real-time monitoring and control [6]. Efficient DT communication relies on technologies such as IoT, standardized information-sharing protocols, and cloud computing, which facilitate seamless data exchange and enable capabilities like virtual commissioning and remote monitoring.

### 2.2. Overview of Existing Digital Twin Review Publications

Research on DT has been reviewed from a broad range of perspectives, covering both cross-industry applications and domain-specific implementations in manufacturing. Early survey studies were primarily oriented toward architecture, enabling technologies, and system-level functionalities of DT systems across multiple sectors [9,10,11]. Several reviews investigated DT frameworks within the Industrial IoT, in which data management, connectivity mechanisms, and multi-domain applications in logistics, transportation, and general industrial environments were examined [9,11]. Although these studies provided foundational insights into DT architectures and data-enabling technologies, their analyses remained domain-agnostic and did not address the specific role of AI in manufacturing-oriented DT systems.

A second group of review studies has focused explicitly on DTs for manufacturing. These works have emphasized the contributions of DTs to smart factory operations, shop-floor monitoring, product lifecycle management, and cyber–physical system integration [10,12,13,14]. Reviews on sustainable intelligent manufacturing have described DT-based approaches for energy optimization and resource-efficiency improvement [12], whereas other studies examined DT applications across design, manufacturing, and maintenance stages [13,14]. Additional domain-specific efforts, including reviews on laser additive and subtractive manufacturing as well as digital-factory implementations, have illustrated how DTs have been adopted to support process modeling and equipment-behavior prediction [15]. Although these studies provide valuable coverage of DT use in manufacturing, their primary emphasis lies in architectural modeling, lifecycle integration, or process-specific implementations. They therefore offer limited discussion of how AI techniques can enhance prediction, optimization, or decision-making within DT-enabled manufacturing systems.

A smaller but increasingly significant body of literature has addressed the integration of AI with DTs. Several surveys investigated AI-enabled DT applications in Industry 4.0, with emphasis on smart manufacturing, industrial robotics, and cyber–physical automation [16,17,18,19,20]. Other works focus on the role of AI in additive-manufacturing DTs, discussing how machine-learning models support defect detection, process-parameter optimization, and quality assurance [21]. Recent reviews examined optimization-oriented AI and DT integration, deep-learning-based DT monitoring, and computer-vision-enabled DT applications in manufacturing operations [18,22,23]. Despite these contributions, AI-DT surveys generally approach the topic from an algorithmic or application-domain perspective rather than examining their roles within manufacturing system layers.

Recent review studies have also examined generative AI within AI-based DT systems [24,25]. These works discuss how generative models support fault diagnosis, predictive maintenance, and virtual process simulation in Industry 4.0 and 5.0 [24]. They additionally outline potential roles of generative techniques in future Industry 6.0 environments [25]. While these reviews summarize emerging generative AI applications in DT research, they do not analyze these methods in relation to manufacturing system hierarchies, indicating the need for an integration-level perspective.

To address these gaps, the present review synthesizes AI-enabled DT applications in manufacturing through a dual axis framework that combines manufacturing hierarchy (machine, cell, shopfloor, and enterprise) with functional categories such as monitoring, prediction, optimization, control, and implementation. This integration-level taxonomy clarifies how individual AI techniques contribute across different hierarchical-levels of manufacturing systems. For this review paper, peer-reviewed publications were collected from Scopus, Web of Science, and Google Scholar using keyword combinations such as “digital twin,” “digital twin using Artificial Intelligence,” “machine learning,” and “smart manufacturing”. The search covered the period from 2017 to 2025.

## 3. AI Methodologies Enabling Digital Twins

As discussed in the previous section, the key distinction between DTs and traditional simulation models lies in their ability to evolve autonomously and reflect real-time operational dynamics. These capabilities are primarily enabled by the integration of AI, which allows DTs to process continuous data streams, adapt to environmental changes, and provide predictive insights beyond static simulations [26].

In this section, core AI methodologies employed in DT systems are introduced and classified according to their learning paradigms and functional roles. Supervised learning has been applied for predictive modeling and classification tasks. Unsupervised learning has been used for pattern discovery and anomaly detection. DL algorithms utilizing multilayer neural networks have been employed to extract hidden information from complex datasets such as images and time series. Reinforcement learning has been adopted for control optimization and sequential decision-making. Finally, hybrid and physics-informed approaches have been utilized to enhance generalization and model fidelity. Specific industrial implementations and application cases are presented in the following section.

### 3.1. Supervised Learning

Supervised learning is a machine learning approach that establishes a functional mapping between input features and labeled outputs by minimizing a task-specific loss function through iterative optimization, enabling accurate prediction on previously unseen data [27].

In the context of DTs, supervised learning has been employed for predictive modeling, classification, and regression tasks [28,29]. Typical applications include fault diagnosis [30], remaining useful life (RUL) estimation [31], and product quality prediction [32]. These tasks are particularly relevant in manufacturing, where large amounts of labeled sensor data are often available from production lines or historical logs. Common algorithms including decision trees, random forests, and convolutional neural networks have been integrated into DT frameworks to enable decision support, early warning, and real-time monitoring functions [17]. To enhance adaptability under variable working conditions in manufacturing settings, combining supervised learning-based DT decision models with transfer learning has been proposed. This approach allows efficient model updates without extensive retraining from scratch, significantly reducing the need for additional datasets. It also minimizes production disruption while maintaining high prediction accuracy [33]. Continual learning strategies have also been developed to dynamically adapt DT models to non-stationary data streams and concept drift. For example, TWIN-ADAPT incorporates online learning within a DT framework, enabling real-time anomaly classification with improved accuracy under evolving industrial conditions [5].

### 3.2. Unsupervised Learning

Unlike supervised learning, which needs labeled data to map input features to output labels, unsupervised learning does not require labeled data. This feature makes unsupervised learning especially useful when labeled datasets are hard to obtain or too expensive to create. Instead of relying on labels, models in this approach discover hidden structures in the data by finding hidden patterns or feature representations [27].

One example is the use of autoencoder-based anomaly detection. Even when trained on unlabeled data, these models can detect minor process abnormalities, including in cases where normal data far outweighs abnormal examples. By generating synthetic abnormal data using DTs, it has been shown that these models can enhance robustness and accuracy in detecting production anomalies in high-value, data-scarce manufacturing processes such as semiconductor bonding lines [34]. Unsupervised methods are also used for such tasks as anomaly detection, clustering different operating states, and compressing features for monitoring and diagnostics. Techniques such as autoencoders and Principal Component Analysis (PCA) have been recently applied in edge-computing setups, allowing real-time fault detection and classification with minimal data while keeping the computations efficient [35,36]. These approaches not only help preserve diagnostic performance with limited resources but also emulate DT functionalities such as virtual fault simulation, adaptive diagnostics, and low-latency inference.

More recently, unsupervised learning has been combined with self-supervised pretraining, representation learning, and hybrid approaches to make the extracted features more relevant and interpretable in real-world industrial settings. For example, vision-based monitoring systems now use autoencoder embeddings and spatial activation mechanisms to track production progress while also detecting anomalies, filtering out visual noise from complex assembly environments [37]. Another example is a predictive maintenance framework built on Contrastive Predictive Coding (CPC), which was applied to roll-to-roll chemical vapor deposition (R2R CVD) processes. By combining data from multiple sensors, this approach was able to predict failures and improve maintenance scheduling without using labeled datasets [38].

### 3.3. Deep Learning

DL is a modeling approach that uses multilayer neural networks, where information passes through a series of nonlinear transformations across hidden layers to generate predictions [39]. In DT systems, DL is especially useful for handling complex and high-dimensional data, such as video, sensor signals, and text. Using these techniques, DTs can perform real-time object recognition, analyze system behavior, and monitor operating states, all of which help support predictive and preventive decision-making in human–machine collaboration [40].

One common application of DL in DTs is surface defect detection under real-time manufacturing conditions. For example, an Edge–Cloud framework has been proposed that processes 2D and 3D data streams and applies a morphology-guided DL model to improve the accuracy of detecting small surface flaws [41]. Object detection methods such as the You Only Look Once (YOLO) algorithms are also widely used. A well-known case is YOLOv3, which was applied in a tool wear monitoring system to classify cutting tools into different wear levels using a labeled image dataset, supporting predictive maintenance of machining tools [42].

Despite their effectiveness, traditional DL models have limitations, especially when dealing with long-range dependencies or combining data from multiple sources. To overcome these issues, Transformer-based models such as Vision Transformers have been developed [43]. In one study, Vision Transformers were used for quality prediction in product assembly under a DT framework. By combining data features including geometry, material properties, and process conditions into a single parameter space, the model was able to predict assembly performance with high accuracy. This approach not only improved reliability but also reduced simulation costs, proving its potential in zero-defect manufacturing [44].

Recent studies show that generative AI can be incorporated into DT-based manufacturing systems to support product and process design. In early-stage applications, large language models (LLMs), particularly ChatGPT versions 3.5 and 4.0, have been employed to generate alternative design pathways and identify potential issues during initial product development [45]. Cognitive DTs have also been combined with generative AI in manufacturing contexts. For example, one study connected a generative AI model (ChatGPT) to a discrete-event simulation-based DT of a production facility via the Node-RED platform, enabling the model to produce logical responses using data supplied by an IoT server [46]. Another approach incorporated a generative AI model into DT-based design-for-manufacturability workflows. In this case, a software environment was developed in which sensor data were used to reproduce a product within a DT for simulation, and a generative AI component generated alternative design solutions based on user requirements and market information [47].

### 3.4. Reinforcement Learning

Reinforcement learning (RL) is a machine learning approach where a model called an agent learns how to make optimal decisions by interacting with its environment and receiving rewards for the taken action instead of relying on labeled data [48]. In DT systems, RL has become an important tool as it can support intelligent decision-making and adaptive control in such areas as manufacturing, robotics, and autonomous systems [49].

With the rise of deep reinforcement learning (DRL), DTs are now able to solve more complex control problems using algorithms such as Deep Q-Networks (DQN), Proximal Policy Optimization (PPO), and Trust Region Policy Optimization (TRPO) [17]. These algorithms can be trained in simulated digital environments using DTs, which makes it possible to explore different scenarios safely, optimize performance under uncertainty, and then apply what is learned to real-world systems. For example, DQN has been used in a virtual aluminum rolling mill to plan and schedule processes, helping the system adapt to changing production conditions [50]. In another study, PPO was applied to train a robotic arm in a Unity-based DT simulation. By using curriculum learning and stable training methods, the agent could successfully learn object manipulation and transfer that skill to a physical robot [51].

To close the gap between simulation and real-world performance, multi-stage training pipelines have been utilized. These usually start with low-fidelity simulations, transition to high-fidelity digital environments, and finally utilize physical hardware [52]. A good example is the training of RL policies in a Unity-based simulator, followed by testing in a software-in-the-loop (SIL) setup, and finally deploying them to a Warthog unmanned ground robot with performance refined at each stage [53]. As DTs continue to grow more complex and connected with real-time control, RL-based methods are evolving toward more efficient, flexible, and transferable learning frameworks.

### 3.5. Hybrid AI Approaches

Hybrid modeling approaches combine data-driven machine learning methods with domain knowledge, physical models, or rule-based heuristics [54]. These methods are used to make models easier to interpret, more generalizable, and more reliable, especially when data is noisy or limited [55]. In DT systems, hybrid models often merge historical sensor data with first-principles equations, which helps improve system prediction and control [56]. By adding physical constraints into the learning process, DTs can maintain consistency with known physics while still adapting to real-time conditions [56].

One area that has gained a lot of attention recently is Physics-Informed Machine Learning (PIML). This approach is considered as a promising alternative to both pure physics-based simulations and conventional data-driven methods, especially for complex applications such as metal additive manufacturing [57]. A well-known type of PIML is the physics-informed neural network (PINN). In this method, physical laws are incorporated into the loss function often in the form as partial differential equations (PDEs) during training [58]. This allows the network to learn system dynamics in a way consistent with physical principles, even when experimental data is limited. For example, it has been shown that PINNs can successfully model complex thermophysical processes in 3D metal additive manufacturing by embedding conservation laws such as momentum, mass, and energy in the neural network. This has enabled accurate predictions with very little experimental data [59]. PINNs have also been extended to prognostics tasks in DTs. In these cases, lower-fidelity physical knowledge, such as monotonicity or curvature constraints, is incorporated into the optimization process. This resulted in higher prediction accuracies and more robustness in situations where detailed physical models are unavailable and data is scarce [60].

### 3.6. Summary

Supervised learning provides strong predictive performance when labeled data are available, but its adaptability can be limited under changing operating conditions without transfer or continual learning. Unsupervised learning avoids the need for labels and is effective for anomaly detection, although the resulting representations may lack interpretability and stability. Deep learning achieves high accuracy in complex perception tasks but requires substantial computation and large datasets; generative DL further supports design exploration and DT-human interaction but remains at an early stage of adoption in manufacturing. Reinforcement learning enables adaptive control but often faces challenges related to training efficiency and simulation-to-real (S2R) transfer. Hybrid and physics-informed approaches improve physical consistency and robustness under limited data but involve higher modeling complexity. These distinctions indicate that no single method dominates across all manufacturing scenarios, and significant gaps remain in scalability, interpretability, and cross-layer DT integration. Table 1 provides a consolidated summary of the AI methodologies discussed in Section 3.1, Section 3.2, Section 3.3, Section 3.4 and Section 3.5, emphasizing their primary manufacturing applications and representative studies.

## 4. Applications of AI-Driven DTs in Manufacturing

AI-driven DTs are transforming manufacturing by enabling predictive, adaptive, and autonomous decision-making across operational scales. From individual machine-level control to enterprise-wide coordination, AI integration extends DT capabilities from simple mirroring to proactive optimization, reducing downtime, improving product quality, and enhancing responsiveness to market dynamics. This section presents the applications and real-world deployments of AI-integrated DTs in manufacturing, organized from the machine level through the cell, shop floor, and enterprise levels.

### 4.1. Machine-Level Digital Twin Applications

At the machine scale, DTs create high-fidelity, real-time digital counterparts to individual manufacturing assets. These implementations continuously monitor equipment status, analyzing wear and damage, predicting performance degradation, and forecasting operational errors. As a result, DTs enable early detection and mitigation of potential failures, minimizing downtime and extending equipment lifespan.

#### 4.1.1. Advanced Machines

DTs play a significant role in enhancing CNC machining processes through real-time monitoring, and operational optimization. Many CNC machines are inherently compatible with DT integration, simplifying bidirectional data exchange by offering adaptable controls, relying on built-in sensors for regular operation, and supporting standardized communication protocols like MTConnect [61,62]. Embedded sensors such as spindle speed encoders, tool position sensors, and feed rate monitors, can be supplemented with basic IoT applications for state determination, enabling DTs to provide continuous insights into machine conditions and performance [61].

DTs replicate the performance of physical systems, accurately simulating tool paths and milling operations. Williams et al. [62] demonstrated this capability through their implementation for Tormach CNC machines(Manufacturer: Tormach; Location: Madison, WI, USA), which replicated physical manufacturing operations by accurately generating tool path positional values (X, Y, Z axes) and capturing live data such as spindle speed via the MTConnect communication protocol. This setup facilitated simulation-based optimization and enabled early anomaly detection by comparing simulated and real-world outcomes. Software platforms such as Siemens Tecnomatix Process Simulate enable the creation of detailed virtual environments for offline robot programming, collision detection, and reachability validation [4]. To ensure simulation fidelity, techniques such as Dynamic Time Warping (DTW) techniques enable rigorous comparison of simulated versus actual tool path data by computing optimal alignment between time-series measurements of machine positions, even when operational timing variations exist [62].

While core machine operations are automated, ancillary processes such as chip removal, are often still performed manually. AI-enhanced DTs close this automation gap by integrating collaborative robots (cobots) and advanced machine vision systems. For example, YOLOv8-based vision models, trained in both simulated and real-world environments, have been applied to optimize chip removal and air-blowing directions using synthetic data and human expertise captured through virtual reality [63]. These models can optimize tasks, close critical automation gaps and improve overall process efficiency.

#### 4.1.2. Predictive Maintenance at Machine Level

**Figure 2:**
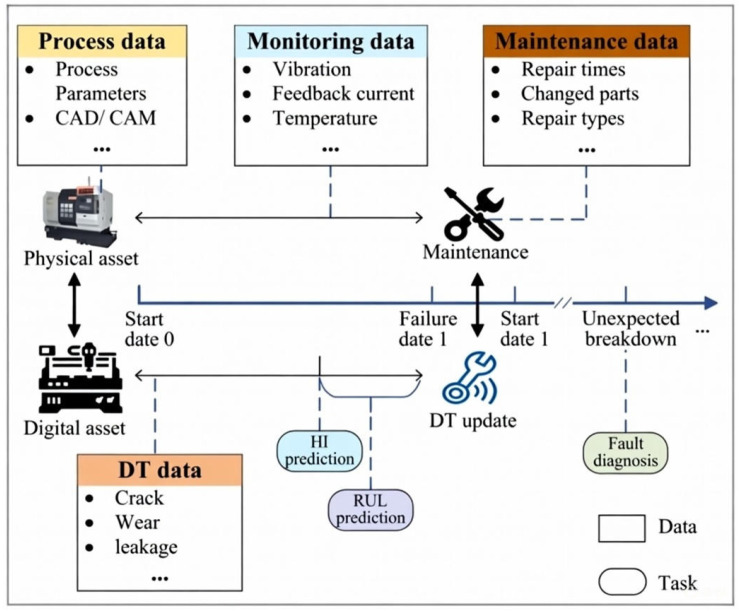
Machine-level DT predictive maintenance framework [64].

The emergence of Industry 4.0 has transformed maintenance strategies for individual machines, shifting from reactive and preventive approaches toward data-driven, predictive maintenance using DTs. This new paradigm, as illustrated in Figure 2, extends conventional condition-based maintenance by relying on continuous equipment monitoring to evaluate critical status metrics, such as health indicators and RUL, for more informed decision-making [64]. Unlike reactive maintenance, which leads to unexpected downtime, or preventive maintenance, which can result in unnecessary part replacement, predictive maintenance optimizes both uptime and resource usage [65,66].

The foundational concepts of DT-based predictive maintenance emerged from aerospace applications, where Glaessgen and Stargel [67] envisioned high-fidelity virtual replicas that combined physics-based simulations with real-time feedback for structural integrity monitoring. Early implementations by Grieves et al. [6] simulated structural fatigue in aircraft fuselage panels under cyclic loading, mirroring physical tests to observe crack propagation and schedule preventative inspections. Tuegel et al. [68] expanded this approach through comprehensive DTs enabling multiphysics, multiscale simulations that incorporated sensor updates and fleet history to forecast health, RUL, and crack propagation. These aerospace-derived principles have since been adapted to manufacturing machine maintenance.

Sim et al. [2] advanced this foundation by developing a DT capable of predicting movements and cutting loads for CNC machine tools with high accuracy, based on integrated models of the controller, feed-drive, and cutting forces. Their parallel development of a condition-monitoring DT for real-time spindle health tracking, using wireless sensors and signal-processing algorithms, demonstrated accurate identification of tool wear progression during CNC operation, showing direct improvements in maintenance scheduling and tool usage efficiency.

#### 4.1.3. Edge Computing and Real-Time Control

Edge computing architectures, where data processing occurs locally on devices near the physical equipment rather than in remote cloud servers, have been proven critical for machine-level DT implementations requiring immediate operational response. Roberto et al. [69] reported a successful implementation of a DT-based edge AI solution for rotating machinery, where the DT collected vibration signals and fed them to lightweight neural networks running on local edge devices. This enabled near-instant fault detection without the latency of cloud computation, providing strong validation for combining edge computing with DTs in latency-sensitive environments.

#### 4.1.4. Generalized Insights

Machine-level DTs fundamentally transform equipment from passive production assets into self-aware, predictive systems. By exploiting existing sensor infrastructure and standardized communication protocols, they enable manufacturers to shift from scheduled maintenance paradigms to continuous condition monitoring with minimal additional investment. The critical architectural requirement is edge computing—processing data locally rather than in the cloud—which proves essential when real-time operational decisions directly impact product quality and equipment availability. Beyond mere monitoring, these DTs close the gap between automated core processes and manual auxiliary tasks through AI-enhanced perception and collaborative robotics, extending intelligent control across the complete production cycle. This pattern, refined from aerospace structural health monitoring, demonstrates that effective machine-level intelligence requires both high-fidelity simulation validation and immediate local processing capability.

### 4.2. Cell-Level Digital Twin Applications

Cell-level DTs represent interconnected groups of machines, robots, or workstations that require coordinated operation. At this scale, DTs extend beyond individual asset monitoring to orchestrate multi-machine workflows, optimize production sequences, and manage resource allocation within manufacturing cells. They are particularly effective in Flexible Manufacturing Systems (FMS), where resources and workflows are dynamically adjusted based on production demands and system constraints. Ullah et al. [70] demonstrated these capabilities in FMS environments, achieving 14.53% productivity gains and 33% improvements in Overall Equipment Effectiveness (OEE) through DT-enabled real-time monitoring and optimization.

#### 4.2.1. Human–Robot Collaboration

The transition to smart manufacturing has significantly accelerated the adoption of industrial robots within manufacturing cells for hazardous and routine tasks, such as transporting heavy materials and performing basic spot welding, thereby freeing up skilled workers for operations that require greater craftsmanship and critical thinking [71]. While these robotic systems offer strong performance repeatability, they often lack awareness of their surroundings, limiting their adaptability in suboptimal or unforeseen operating conditions. DTs address these limitations by enabling the development and deployment of intelligent robotic agents for autonomous and collaborative tasks.

**Figure 3:**
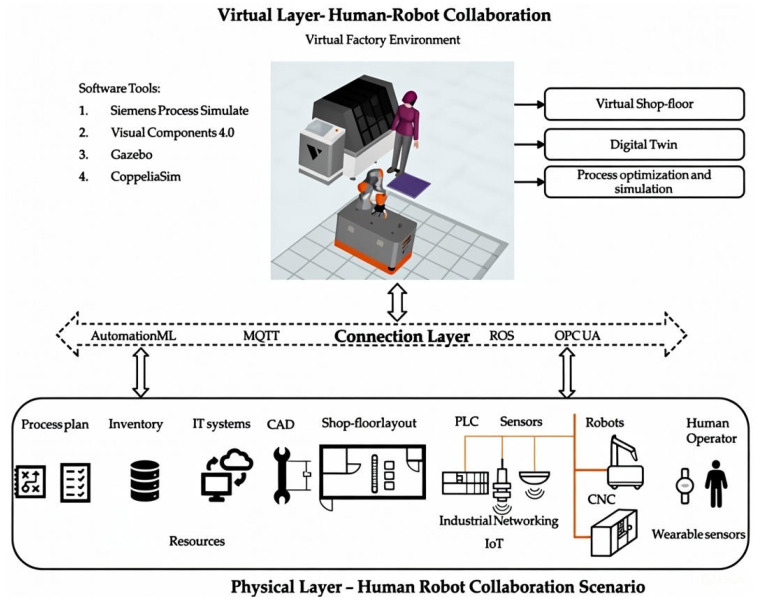
DT-based human–robot collaboration [73].

DTs play a critical role in enhancing safety and efficiency within automated manufacturing cells by enabling detailed simulation of human–robot interactions and supporting comprehensive scenario testing that improves the reliability of collaborative processes. They facilitate dynamic task delegation in human–robot collaborative assembly, continuously optimizing how each agent’s strengths are utilized [72]. In Human–Robot Collaboration (HRC) environments, where physical barriers are often impractical, the DT architecture shown in Figure 3 integrates AI-enabling technologies across layered physical, connection, and virtual components to address safety and coordination challenges. The physical layer employs machine vision for real-time human detection and tracking, IoT sensors to monitor robot states such as velocity, torque, and position, and wearable sensors to capture operator movements. The connection layer uses automated machine learning pipelines to process this multisensor data, enabling predictive safety monitoring and adaptive decision-making through communication protocols including MQTT, ROS, and OPC UA. The virtual layer leverages simulation platforms such as Visual Components, Gazebo, and CoppeliaSim to train and validate AI models in a risk-free environment prior to deployment. Collectively, this layered DT framework supports intelligent functions such as real-time collision prediction, dynamic task reallocation, and adaptive safety-zone adjustment [73].

In the long term, the adaptability of DTs across the robot’s lifecycle ensures that the robot’s capabilities evolve in tandem with the operator’s growing skill set, supporting optimal collaboration at every stage [73]. Bilberg and Malik [72] demonstrated how DTs facilitate task delegation in collaborative assembly, where the system continuously learns optimal task distribution between human operators and robots based on their respective strengths and current performance metrics.

#### 4.2.2. Quality Control Cells

DTs inherently support quality control through robust predictive and prescriptive capabilities at the cell level, significantly enhancing defect detection and prevention. By leveraging advanced machine vision and simulation capabilities, DTs can validate product performance at early stages and detect potential faults before they propagate downstream. This early detection allows for timely corrective action through autonomous virtual-to-physical communication, reducing rework, material waste, and quality variation [74]. Building on foundational DT implementations [62], advanced temporal intelligence applications use LSTM neural networks to forecast system states based on historical sensor data, enabling quality predictions by capturing how system behavior changes over time. This approach allows cell-level DTs to simulate possible future scenarios and anticipate quality deviations before they occur, supporting proactive quality management.

DT predictions can also be used to provide quality control strategy recommendations towards zero-defect manufacturing, enabling flexible and efficient responses to quality issues as they arise [75]. Continuous monitoring and advanced process modeling enable the most cost-effective combination of predictive and reactive quality control approaches to be tailored to each stage of production, while accounting for case-specific product and demand characteristics [75]. By integrating quality control directly into cell workflows, DTs ensure that quality assurance is not a post-production step but a continuous, adaptive process.

#### 4.2.3. Virtual Commissioning

A crucial component of cell-level DT implementation is virtual commissioning, the process of simulating and validating automation control logic in a virtual environment before physical deployment, which accelerates the testing and validation of advanced control systems, especially in complex robotic manufacturing cells. DT simulations provide an efficient environment to test and fine-tune control systems prior to deployment in physical systems [71]. This approach significantly reduces implementation risks and debugging costs, facilitating rapid iteration and control logic refinement before deployment. By eliminating delays tied to hardware availability during the development phase, virtual commissioning significantly shortens the overall system deployment timeline [71].

#### 4.2.4. AI-Driven Agent Training

The connectivity and decision-making capabilities of AI-integrated DTs enhance the resilience of robotic cells through agent training and deployment. Agent training involves using virtual environments to teach AI systems optimal decision-making behaviors through iterative learning processes. DTs serve as robust platforms for the training, validation, and testing of DRL agents in virtual environments [4,76]. Han et al. [77] demonstrated this approach by developing a DT environment that could train autonomous robots through reinforcement learning before real-world deployment. Their framework simulated environment dynamics and robot interactions, using convolutional neural networks for sensor processing to enable dynamic trajectory adaptation, effectively transforming the DT from a passive monitor into an active trainer and optimizer. These virtual agents, called “Digital Engines,” can support complex functions such as dynamic scheduling tasks [4] and multi-robot collaborations, ensuring that intelligent behaviors are refined prior to real-world implementation.

#### 4.2.5. Generalized Insights

Cell-level DTs coordinate multiple machines and robots to achieve capabilities that individual assets cannot. Unlike machine-level DTs that monitor single assets, cell-level systems orchestrate workflows, allocate resources, and optimize interactions between multiple physical entities. The key enabling technology is predictive modeling combined with AI integration, which transforms reactive systems into proactive ones. The virtual layer serves two critical functions: validating control logic and safety protocols before physical deployment, and training AI agents through iterative learning in risk-free environments. This dual function represents a shift from using DTs as verification tools to using them as optimization platforms. Across all cell-level applications including human–robot collaboration, quality control, virtual commissioning, and agent training, the primary objective is preventing errors from propagating downstream, achieving localized resilience that protects the broader manufacturing system from cascading failures.

### 4.3. Shop Floor-Level Digital Twin Applications

At the shop floor scale, DTs form system-of-systems architectures, integrating machines, cells, and human operators into a cohesive cyber–physical production environment. The paradigm of the DT shop floor (DTSF), proposed by Tao et al. [7], envisions total cyber–physical integration of a manufacturing line using DTs. This system-of-systems-level application advances the Industry 4.0 objective of decentralization through localized and in some cases automated decision-making. DT services at this level include continuous process optimization [22], optimization of shop floor layout [78], and virtual process planning and commissioning [79].

#### 4.3.1. General Architecture of DT-Based Shoop Floor

**Figure 4:**
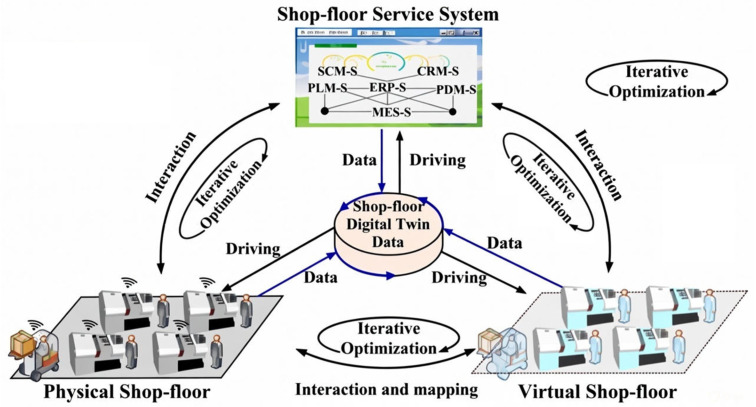
Conceptual model of DT-based shop floor [7].

Figure 4 illustrates the four-component architecture of a DT-based shop floor, where AI integration enables intelligent coordination across physical and virtual manufacturing environments [7]. In this architecture, the Physical Shop-floor layer—comprising human operators, equipment, materials, and environmental conditions—is instrumented with IoT sensors that continuously generate real-time operational data such as position, speed, temperature, energy usage, and operator actions. This data feeds into the Shop-floor Digital Twin Data (SDTD) component, an AI-enabled integration hub that aggregates sensor streams, virtual model outputs, and enterprise information while applying machine learning algorithms for pattern recognition, anomaly detection, and predictive analytics through bidirectional real-time data flows. The Virtual Shop-floor layer provides high-fidelity simulation environments where reinforcement learning agents can be trained safely and where physics-informed and computer-vision-based neural networks refine model accuracy through continuous calibration. Complementing these layers, the Shop-floor Service System (SSS) delivers AI-driven functions through modules for automated data preprocessing, intelligent scheduling and resource allocation, predictive maintenance, and adaptive process control. Together, these components form a continuous learning loop in which physical data train AI models, optimized strategies are validated virtually, AI services implement decisions on the physical floor, and resulting outcomes feed back to improve model performance.

Building on individual machine implementations, Tao’s framework scaled this concept to a full factory floor, integrating multiple machines through interconnected communication architectures [7]. This implementation demonstrates that combining local AI models with centralized cloud-based intelligence enables dynamic resource allocation and production forecasting, introducing the concept of “multi-twin synchronization” and system-level intelligence foundation for smart factories. This system-wide deployment was further expanded by Tao et al. [80], who linked multiple production units coordinated by a cloud-based control center with distributed AI capabilities, proving that DTs could scale beyond isolated machines to govern entire production ecosystems.

#### 4.3.2. Bottleneck Detection and System Efficiency

Overall equipment efficiency metrics are a major maintenance concern in manufacturing shop floor systems, for which calculations can be simplified with DT technology [81]. Bottlenecks are a critical indicator of inefficiency in a manufacturing system, and shop floor-level DTs can be applied towards their identification and mitigation [82]. Constant monitoring through synchronized physical and digital production system operation enables rapid anomaly detection, highlighting opportunities for physical process improvement and DT calibration [7].

The practical impact of system-wide DT monitoring has been empirically demonstrated in large-scale industrial implementations. Daraba et al. [83] applied shop floor-level DT monitoring in industrial printing operations, achieving measurable gains in production efficiency and predictive maintenance scheduling across their 150-machine installation. This system collected data on the relevant parameters of machine operation and tracked performance to provide actionable insights. The real-time monitoring through DTs resulted in a 10% reduction in production time, demonstrating the practical value of DTs in continuously optimizing machine utilization, resource allocation, and overall process efficiency. This comprehensive DT solution also enhanced predictive maintenance by enabling early detection and handling of potential issues, thereby significantly reducing machine downtime and operational costs in alignment with Industry 4.0 objectives.

The machine-to-machine communication enabled by DTs can create a dynamic, responsive shop floor capable of adapting to the constantly shifting priorities and optimization objectives of a manufacturing environment. Such DT applications lay the foundation for highly efficient, fully integrated smart factories. Moving towards total supply chain collaboration through vertical and horizontal DT integration can foster interconnectivity and sustainability across all levels of production [84,85].

#### 4.3.3. Flexible Manufacturing Systems

A popular response to increasing market volatility is flexible manufacturing. This approach requires frequent shop floor resource reallocation that is simplified with DT data-based decision making. Manufacturing process reconfiguration is made possible through AI integrated DT optimization tactics, promoting autonomous response to fluctuations in demand [78]. Shop floor-level DTs provide strategic resource management tools, including real-time location and availability data that can be used to simulate efficient and collision-free transitions [79,86].

In addition to shop floor layout optimization, DTs can support the adaptability required for modern manufacturing by coordinating the optimization of individual equipment states in real-time towards greater overall shop floor efficiency [87]. Reconfigurable machine tools enable flexible manufacturing without the need for time-consuming and potentially hazardous rearrangement of heavy equipment. Using real-time tool condition and availability data from individual machines, DT-driven optimization during process planning can reduce the collective number of tooling reconfigurations required for the shop floor, further increasing the efficiency of FMS [87].

Furthermore, from a process design perspective, integrating product and process DTs enables product customization at the unit level. Onaji et al. [88] demonstrated this approach using RFID technology, allowing each work-in-progress item to communicate its specific production steps directly to manufacturing equipment via their respective DTs at every stage of production.

#### 4.3.4. Virtual Commissioning and Process Planning

Virtual commissioning leverages DT technology to validate production configurations in risk-free digital environments before physical implementation, significantly reducing deployment time and operational disruption. BMW has demonstrated this approach at scale using NVIDIA’s Omniverse platform to create fully virtual assembly lines for layout validation, worker training, and robotic path planning without disrupting active production [89]. This virtual commissioning methodology allows comprehensive testing of new production configurations, equipment interactions, and human–robot collaboration scenarios before physical implementation, significantly reducing deployment risks and optimization time.

The successful implementation of such large-scale virtual environments requires robust technological foundations. Fuller et al. [26] provided a technological roadmap for scalable shop floor DT systems, surveying enabling technologies including sensor fusion, cloud computing, and system integration frameworks necessary for comprehensive virtual commissioning capabilities. Broader literature emphasizes that interoperability remains critical for seamless integration across multiple DTs operating at different hierarchical levels [30,90]. Schroeder et al. [91] complemented this work by proposing systematic methodologies for DT modeling in industrial automation processes, focusing on how DTs can integrate sensor data and historical process logs to enable monitoring, prediction, and optimization of industrial system behavior within Industry 4.0 contexts. These foundational frameworks enable virtual commissioning to serve as a bridge between design and deployment, allowing manufacturers to iteratively refine production systems in the digital realm before committing to physical changes.

#### 4.3.5. Process Optimization and Control

Optimization in manufacturing presents a unique challenge as decision-makers seek to balance multiple, often conflicting objectives, including cost, safety, sustainability, and quality, all within stringent design and resource constraints. DTs facilitate dynamic optimization in manufacturing by reflecting real-time system conditions and adapting control strategies for enhanced production performance and reduced operational costs. DT-driven process optimization can be implemented either online or offline depending on the urgency of the application [22]. The real-time adaptive capabilities of online methods come at the expense of increased computational requirements and, in some cases, model instability that are of less concern with offline approaches.

A prominent example of shop floor optimization is the “Digital Engine” concept proposed by Xia et al. [4], which can be scaled up from the manufacturing cell level to illustrate how AI-driven DTs efficiently acquire process knowledge, optimize scheduling, and identify optimal actions in real-time, significantly improving workflow coordination and production throughput. Digital Engine uses reinforcement learning to continuously improve decision-making based on production outcomes, creating an adaptive system that learns from experience.

Resources including equipment and personnel can be optimally distributed based on real-time feedback to minimize downtime and reduce equipment deterioration rate through availability and strength-based task delegation [92,93]. Yan et al. [93] demonstrated how double-layer Q-learning algorithms integrated with DTs enable dynamic scheduling with preventive maintenance, significantly reducing unexpected failures while optimizing production sequences.

#### 4.3.6. AI-Driven Analytics Integration

Rathore et al. [76] provide a systematic review of AI, machine learning, and big data integration in shop floor DTs, summarizing methods such as Support Vector Machines (SVMs), Bayesian Networks, random forests, ANN models, and DL approaches for maintenance forecasting and fault diagnosis. Qi et al. [94,95] operationalized these concepts by integrating big data analytics with DTs to develop predictive models for complex manufacturing equipment, enabling real-time adjustment of operating conditions to extend component lifespans through enhanced predictive maintenance and optimization [96].

#### 4.3.7. Generalized Insights

Shop floor-level DTs function as system-of-systems frameworks that unify machines, cells, human operators, and enterprise-level data into an integrated cyber–physical environment capable of real-time learning, coordination, and optimization. Their defining characteristic is the ability to transform the shop floor from a static production space into a dynamic, data-driven ecosystem where distributed AI agents continuously monitor operations, detect bottlenecks, forecast disruptions, and autonomously adjust processes. Core capabilities include high-fidelity virtual simulation for training and validation, AI-enabled scheduling and resource allocation, predictive maintenance, flexible manufacturing support, and virtual commissioning for risk-free process planning. These DTs provide measurable benefits such as reduced production time, improved equipment utilization, adaptive reconfiguration in the face of demand variability, and enhanced system-wide efficiency.

### 4.4. Enterprise-Level Digital Twin Applications

At the enterprise scale, DTs integrate design, production, logistics, and lifecycle management into a unified decision-making framework. AI-driven analytics link operational data with business intelligence, enabling predictive maintenance, supply chain optimization, and strategic planning across the organization. Because these systems often span multiple facilities, they rely on standardized communication protocols and cloud-based orchestration to ensure interoperability. As DT technologies mature, they increasingly support enterprise-wide coordination, facilitating strategic decision-making, lifecycle planning, and cross-functional collaboration. These implementations extend DT applications into domains such as systems engineering, sustainability assessment, and digital transformation strategy, positioning DTs not merely as operational tools but as enablers of broader organizational evolution.

#### 4.4.1. General Architecture of Enterprise-Level Integration of DTs

**Figure 5:**
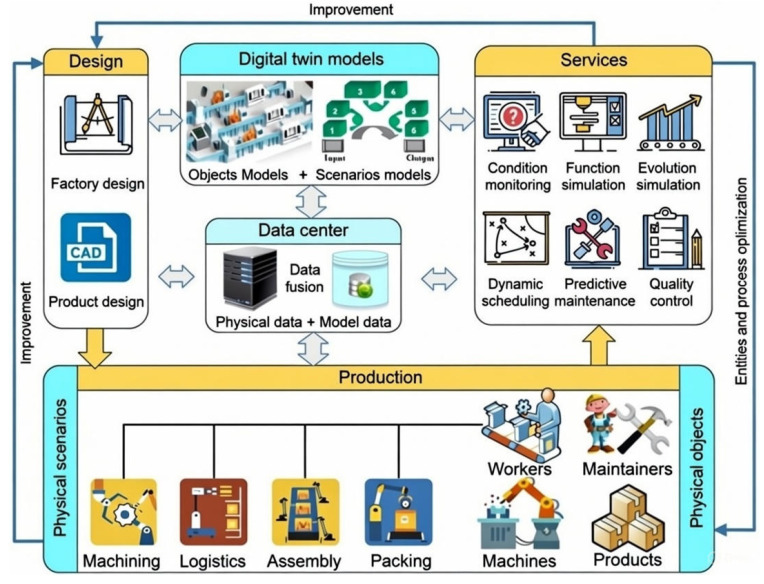
Enterprise-level DT framework [94].

Figure 5 illustrates an example of the enterprise-level integration of DT technologies across design, production, and service domains. The diagram shows how factory and product design feed into DT models, which combine object and scenario representations. These models interact with a centralized data center that fuses physical and model data, enabling advanced services such as condition monitoring, predictive maintenance, dynamic scheduling, and quality control. At the bottom, physical scenarios including machining, logistics, assembly, and packing, are linked to workers, machines, and products, creating a closed loop between physical operations and virtual models. This structure highlights how DTs support continuous improvement and process optimization across the entire lifecycle at the enterprise level.

#### 4.4.2. Systems Engineering and Full Lifecycle Management

DTs have emerged as foundational enablers for comprehensive systems engineering approaches that span entire product and system lifecycles. Madni et al. [97] demonstrated the evolution of DTs through their application as a foundation for model-based systems engineering (MBSE) across aerospace and defense sectors, detailing full lifecycle applications from design through sustainment where DTs simulate, validate, and refine mission-critical components. Their work reveals how DTs serve as central coordination mechanisms for complex engineering programs, enabling comprehensive lifecycle management and mission-critical system optimization. James et al. [98] operationalized this vision through a MBSE approach, using SysML to demonstrate how requirements, architecture, and simulation models can be unified into verifiable DT frameworks that reduce interdisciplinary ambiguity and promote system coherence. Together with foundational works, these established the theoretical and methodological backbone that enables enterprise-wide implementation.

By maintaining continuous data threads from early modeling to post-deployment feedback, enterprise DTs reduce program risks, improve traceability, and support agile redesign cycles throughout complex system development. This practical MBSE implementation addresses real-world challenges such as data integration across design phases, validation of virtual–physical synchronization, and maintenance of model fidelity throughout extended operational lifecycles.

#### 4.4.3. Digital Transformation and Cross-Functional Integration

The strategic value of DTs in digital transformation extends to enabling cross-functional collaboration from production teams to executive leadership through shared digital interfaces. Rasheed et al. [99] present a value-driven approach to DTs by mapping their benefits across operational, strategic, and organizational layers, with case studies in manufacturing highlighting how DTs facilitate cross-functional collaboration. Their research illustrates practical implementations where DTs enable real-time data sharing between design, production, and quality teams, reducing time-to-market for new products while improving first-pass yield rates.

Barricelli et al. [100] support this perspective through their large-scale survey of DT applications, categorizing use cases across manufacturing, aviation, and healthcare sectors while providing meta-analysis on how DTs have diversified across industries. Their work highlights both the opportunities for cross-functional integration and the challenges including seamless data exchange and maintaining high-fidelity virtual models that must be addressed for successful enterprise-wide adoption. DTs shift from being engineering tools to central pillars of digital transformation, enabling organizations to break down silos and create unified decision-making frameworks. This transformation facilitates real-time visibility across all organizational levels, from shop floor operators tracking production metrics to executives monitoring strategic key performance indicators.

Enterprise-level AI integration focuses on strategic decision-making frameworks that connect operational DT data with business intelligence systems, enabling real-time strategic adjustments based on production performance, market demands, and resource availability. Advanced enterprise architectures incorporate machine learning models that analyze cross-functional data streams to optimize not just individual processes, but entire business operations including supply chain coordination, workforce allocation, and capital investment decisions.

Siemens has applied enterprise DTs for workforce retraining, using immersive virtual simulations to transition workers from other industries into manufacturing roles [101,102]. This application demonstrates how enterprise DTs extend beyond operational optimization to address human capital challenges, supporting workforce development and knowledge transfer across the organization. This structured approach to enterprise AI integration positions DTs as critical enablers of intelligent and sustainable industry transformation. They extend beyond shop-floor applications to support enterprise-level design decisions, connect departments through shared digital models, and drive organizational change toward more intelligent and responsive industrial ecosystems.

#### 4.4.4. Digital Twin Networks and Multi-Site Coordination

Schroeder et al. [91] proposed a methodology for DT design and deployment specifically for Industry 4.0, characterized as being flexible and generic, utilizing model-driven engineering (MDE) for DT design. The proposed architecture was demonstrated through a case study involving an oil refinery system, which included elements such as automated valves, an intelligent maintenance system (IMS), and a dashboard for visualization. It was shown that this methodology has the potential for implementation across diverse applications, thereby facilitating the deployment and integration of new services within advanced manufacturing and automation systems.

Yiwen et al. [103] complement this work with a comprehensive survey on Digital Twin Networks (DTNs), which explores how virtual representations achieve co-evolution between physical and virtual spaces through integrated DT modeling, communication, computing, and data processing technologies, identifying manufacturing systems as a key application scenario alongside aviation, healthcare, and smart cities. Tao et al. [80] demonstrated a cloud-coordinated multi-site DT system for predictive maintenance, production planning, and resource allocation, showing scalability from local operations to enterprise-level orchestration. Their implementation connected multiple manufacturing facilities through a unified DT platform, enabling coordinated decision-making across geographically distributed operations while maintaining local autonomy for site-specific optimization. Together, these studies demonstrate how embedding AI into DTs supports predictive capabilities, adaptive control, and learning-based optimization, offering a bridge between traditional automation and fully autonomous manufacturing systems.

#### 4.4.5. Generalized Insights

Enterprise-level DTs are distinguished by three integrative capabilities: (1) lifecycle continuity that maintains data coherence from initial design through decades of operation, (2) cross-functional coordination that connects departments operating on different objectives and timescales, and (3) multi-site orchestration that balances centralized strategy with local operational autonomy. The underlying necessity is straightforward. Strategic decisions such as capital investments, product portfolio choices, and multiyear development programs require integrating information across organizational boundaries, facility locations, and temporal horizons that no single production system can span. While shop floor DTs optimize operations within a facility, enterprise DTs coordinate strategic choices across facilities, functions, and lifecycles where misalignment creates business-level risk rather than operational inefficiency.

### 4.5. Summary

The progression from machine-level to enterprise-level DT applications demonstrates the scalability and versatility of AI-integrated DTs in manufacturing. At the machine level, DTs provide granular monitoring and control of individual assets, achieving measurable improvements in maintenance scheduling and operational efficiency. Cell-level implementations coordinate multiple assets and enable human–robot collaboration, while shop floor DTs integrate entire production systems for dynamic optimization and flexible manufacturing. At the enterprise scale, DTs transcend operational boundaries to enable strategic decision-making, lifecycle management, and cross-functional integration.

The case studies presented across these four hierarchical levels reveal consistent themes: the critical role of AI in transforming DTs from passive monitors to active optimizers, the importance of bidirectional communication between physical and virtual spaces, and the necessity of scalable architectures that can grow from single machines to enterprise-wide deployments. As manufacturing continues its digital transformation, these multi-scale DT implementations provide the foundation for autonomous, adaptive, and intelligent production systems that can respond to the demands of Industry 4.0 and beyond.

## 5. Current Challenges and Limitations

The development of AI-integrated DTs faces several interconnected challenges across technical, operational, and business domains. Overcoming these limitations is essential to realizing the full potential of DT technology in smart manufacturing systems.

### 5.1. Data Quality and Synchronization Issues

One of the primary challenges in DT development is the effective integration and management of data. Managing large volumes of real-time data from diverse sources remains a significant challenge, particularly when ensuring data quality and maintaining seamless bidirectional communication between physical and virtual systems. Discrepancies between physical and digital presentations, along with communication delays caused by hardware latency, can lead to synchronization errors, model non-convergence, and fragmented data structures. Addressing these issues will require advanced strategies for structuring, validating, and synchronizing high-fidelity data to ensure accurate, scalable, and trustworthy DT operations.

### 5.2. Model Fidelity, Training, and Generalization

The performance and effectiveness of AI-integrated DTs depend heavily on accurate modeling and effective training methodologies. Challenges span from basic representation accuracy to complex system integration, directly impacting the reliability, scalability, and practical deployment of DTs in industrial environments.

#### 5.2.1. Virtual-Physical Realism Gap

A fundamental challenge in developing DTs is the “realism gap” between virtual models and their physical counterparts. AI algorithms trained using simulated data rely heavily on the accuracy of the digital representation. When these models fail to capture real-world dynamics—such as sensor noise, actuator delays, nonlinear system responses, or environmental variability—the resulting predictions and control strategies may not transfer reliably to the physical system. This gap is particularly problematic for applications involving complex physical interactions, machine-specific behaviors, or high-precision control, where even small mismatches between simulation and reality can lead to suboptimal or unsafe outcomes.

#### 5.2.2. Training Optimization: The Exploration-Exploitation Dilemma

In training AI models, particularly those based on DRL, the exploration–exploitation trade-off presents a fundamental challenge. This involves balancing two competing objectives: encouraging agents to take new actions to explore optimal solutions, while simultaneously exploiting known strategies to achieve faster convergence and stable performance. This balance is especially difficult to achieve in complex manufacturing systems where state transitions are nonlinear or unpredictable. Excessive exploitation can result in suboptimal learning outcomes, while excessive exploration may slow convergence and increase training time, limiting real-time applicability. Optimizing this trade-off is important for developing robust and efficient DRL-based DTs.

#### 5.2.3. Model Robustness and Generalization

A major advantage of DTs lies in their ability to predict and adapt to “unpredictable, undesirable” behaviors [6]. Achieving this capability requires preventing overfitting and ensuring generalization across diverse operating conditions. To improve model robustness, noisy or low-quality data are sometimes deliberately incorporated into training sets to simulate real-world variability [3]. Even with well-trained models, many DT applications remain highly tailored to specific services, limiting generalizability across broader manufacturing contexts. To address this, transfer learning can help adapt knowledge from specialized DTs to similar tasks, enhancing reusability and scalability [104,105]. Expanding the scope of DT-driven Product Lifecycle Management (PLM) through AI will promote integration across the entire product lifecycle from design to disposal, thereby improving system-wide adaptability.

### 5.3. System Scalability and Interoperability

Scalability and interoperability are essential for deploying DTs across entire manufacturing systems. “Plug-and-play” model design supports these goals by offering standardized platforms that allow users to add or modify DT applications as manufacturing needs evolve [106]. This modularity simplifies machine-to-machine communication and promotes seamless integration across distributed systems.

However, several technical challenges remain. Advancing intelligent perception and connectivity technologies, especially real-time data acquisition and semantic communication between cyber–physical systems, is a major research priority. Software architecture also plays a critical role. While incorporating OEM software may simplify DT construction, proprietary protocols can hinder interoperability. Adopting standardized data formats and I/O protocols can ensure uninterrupted data flow across DT systems and shop floor components. Broader adoption of these standards by OEMs and DT-as-a-service providers is vital for building autonomous, interoperable, and scalable DT ecosystems.

### 5.4. Data Security and Proprietorship

Data security is one of the most frequently cited concerns limiting DT adoption [62,84,107,108]. This is particularly relevant in full product lifecycle applications, where OEMs may be reluctant to share sensitive design and manufacturing data, despite its importance for operation and maintenance. To address this, Mikołajewska et al. [24]. proposed using federated learning to enhance security while enabling collaborative model development. This approach allows organizations to share insights without exposing proprietary data, facilitating innovation while preserving competitive advantage.

In addition to technical safeguards, legal questions around data ownership must also be addressed [109]. Such concerns are particularly prominent in full lifecycle DT applications, for which data generated during early design and production stages are essential for effective operation, maintenance, and decommissioning. Further standardization is needed to determine whether end users have the right to access such data for their purchased systems and under what conditions [110]. Without clear legal guidance, uncertainty over data proprietorship may hinder broader DT adoption, especially in industries where intellectual property is tightly guarded.

### 5.5. Commercial Solutions

Currently, commercial DT solutions are sparsely documented in peer-reviewed publications. Siemens stands out as the only solution provider with extensively reported implementations, spanning applications in oil and gas operations, discrete-part manufacturing, logistics, automotive assembly, and educational laboratory environments [111,112,113,114]. These implementations generally follow one of two approaches: (1) cloud-native microservice architectures designed for brownfield integration capable of consolidating heterogeneous legacy data and supporting photogrammetry, asset information modeling, and lifecycle management, or (2) tightly integrated three-layer DT architectures linking physical equipment, simulation platforms such as Tecnomatix, and real-time synchronization via SIMATIC PLCs and OPC UA. Reported outcomes include improved throughput, reduced diagnostic time, enhanced bottleneck detection, and more structured maintenance workflows. In contrast, PTC technologies appear only in supporting middleware roles such as data aggregation and protocol translation, and no peer-reviewed study documents a complete DT implemented using Dassault Systèmes tools. This concentration of published evidence around Siemens-centric ecosystems highlights a broader ecosystem limitation: organizations may face restricted platform choice, fragmented tool chains, and uncertainty regarding the transferability of DT solutions across different industrial software environments.

### 5.6. Large-Scale Real-Time DT Deployment

#### 5.6.1. Communication Latency and Network Constraints

Communication latency between cloud, edge, and device layers remains a persistent bottleneck in distributed DT architectures. Recent research has shown that maintaining synchronized digital representations across multi-tier networks introduces construction delays and synchronization delays that can critically impact system responsiveness [115]. Studies on DT edge networks have demonstrated that even optimized architectures must carefully balance task offloading decisions to minimize end-to-end latency while managing bandwidth constraints between layers [116]. In industrial settings where fault progression can occur within seconds, network delays of even 100–200 milliseconds during cloud-edge data transmission can compromise the effectiveness of predictive interventions, necessitating sophisticated edge preprocessing and feature extraction to reduce communication overhead.

#### 5.6.2. Data Acquisition and Labeling Limitations

Data acquisition and labeling pose equally significant challenges for industrial DT implementations. Unlike consumer applications with abundant labeled datasets, industrial environments typically operate in normal states for extended periods, creating severe class imbalance where fault samples may represent less than 1–5% of collected data [117]. This scarcity is compounded by the resource-intensive nature of obtaining labeled fault data as actual equipment failures are costly, and deliberately inducing faults for data collection is often infeasible in production environments. Furthermore, industrial data labeling requires domain expertise to accurately categorize fault types, vibration patterns, and degradation states, making the annotation process both time-consuming and expensive. The proprietary nature of industrial processes also limits data sharing between organizations, hindering the creation of large-scale benchmark datasets that have accelerated progress in other AI domains [118].

#### 5.6.3. Safety, Interpretability, and Model Drift

Safety considerations add another layer of complexity to real-time edge DT deployments. Industrial fault detection systems must achieve response times under 50 milliseconds to prevent catastrophic failures, requiring deterministic performance guarantees that go beyond average-case latency metrics [116]. False positives can trigger unnecessary production halts costing hundreds of thousands of dollars per minute, while false negatives risk equipment damage, worker safety incidents, and environmental hazards [119]. The interpretability of AI-driven fault detection becomes critical in safety-critical contexts as operators must understand why the system flagged a particular condition to make informed decisions about intervention versus continued operation. Model drift, where real-world operational data diverges from training distributions, necessitates continuous monitoring and retraining capabilities at the edge, adding operational complexity to deployed systems. These multifaceted implementation challenges underscore that while edge-based DTs offer tremendous potential for industrial applications, their successful deployment requires careful consideration of not only computational architecture but also data infrastructure, safety validation, and operational robustness.

### 5.7. Implementing AI-Driven Digital Twins in Existing Manufacturing Environments

Most real-world DT deployments occur in brownfield settings where legacy machines, heterogeneous controllers, and incomplete data infrastructures pose significant integration challenges. Implementing an AI-driven DT in such environments requires retrofitting existing assets with IoT sensors and edge devices to capture high-resolution operational data without modifying established automation systems. Interoperability is achieved through protocol translation layers—such as OPC UA wrappers, MQTT brokers, and middleware like KEPServerEX—that unify disparate communication standards and expose machine data to DT platforms. Because engineering documentation for older equipment is often limited, hybrid DT models that combine simplified physics-based representations with data-driven AI methods (e.g., LSTM forecasting, anomaly detection, or virtual sensing) are commonly used to approximate system behavior. AI training and validation are typically performed in virtual commissioning environments, allowing reinforcement learning agents or predictive models to be refined safely before deployment. Bidirectional synchronization with the physical system is introduced gradually, beginning with monitoring and advisory functions and evolving toward partial or full closed-loop control as reliability increases. Although brownfield DT implementation is more constrained than greenfield deployment, these incremental and AI-enabled strategies allow manufacturers to modernize existing infrastructure while minimizing disruption to ongoing operations.

## 6. Strategic Opportunities and Research Frontiers

### 6.1. Human-in-the-Loop and Remote Interventions

The changing role of human engagement in increasingly autonomous systems presents both challenges and opportunities. Whereas adaptive intelligence demonstrates strong control capabilities, human operators remain superior in adapting to unforeseen events in complex environments. As a result, future smart factories are expected to increasingly support “cognitive social human-in-the-loop” architectures, where human interventions are reliably incorporated in automation systems, especially in situations with limited prognostic knowledge of unexpected incidents. This vision requires developing secure remote human monitoring and intervention capabilities that align with current industrial practices and protocols.

Remote monitoring is a widely supported feature in many DTs, with some applications progressing towards full remote engagement [71,120]. This capability is reshaping how manufacturing systems are maintained and supervised. However, remote interventions must prioritize safety and exercise caution if eliminating in-person equipment verification prior to interacting with heavy machinery. Such applications must also be robust to cyber threats as malicious tampering could have hazardous physical consequences. This includes ensuring that safety-critical functions are locally controlled while allowing remote access for non-safety-critical operations through platforms like customized OPC clients and IoT interfaces. The overarching goal is to integrate autonomous decision-making with secure remote human interventions, allowing stakeholders to design, simulate, program, and control distributed virtual cells, followed by the remote integration of safely commissioned programs in physical cells.

**Figure 6:**
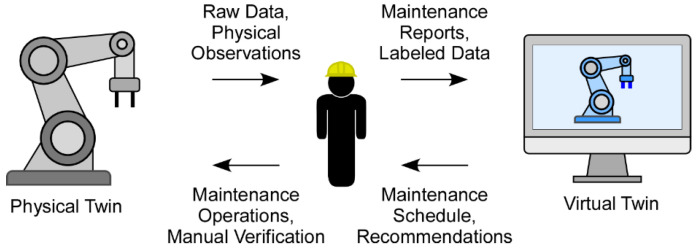
Basic architecture of Digital Twin systems with human-in-the-loop.

Even in some cases where fully autonomous DT-driven corrections are possible, human verification is still retained to implement system recommendations [92]. While this may seem contrary to the automatic data flow envisioned by Kritzinger et al. [8], such safeguards can be an advantageous method to incorporate experienced operator knowledge into the DT decision-making process. This can give manufacturers peace of mind through simple sanity checks that prevent a DT in need of calibration from taking improper action. Moreover, incorporating human expertise into the training loop, such as labeling data or providing feedback on predictions, enhances the robustness and trustworthiness of AI-driven DTs [121]. An example of the role of human intervention in DT system architecture is shown in Figure 6. This collaborative framework ensures that human expertise continues to play a vital role in validating AI decisions and adapting to novel situations, promoting a collaborative ecosystem between humans and intelligent machines.

### 6.2. Hybrid Modeling (Physics + AI)

As DTs evolve to support more intelligent and autonomous manufacturing systems, developing advanced service analysis methods based on DT-generated data will be increasingly important. Sophisticated AI algorithms enable the processing of complex, high-dimensional datasets into actionable insights for optimization and decision-making. However, this advancement towards greater accuracy in DT optimization and prediction often comes at the expense of transparency. The “black-box” nature of many AI techniques can limit trust and hinder adoption among stakeholders, particularly in safety-critical or regulated environments. Therefore, achieving the right balance between accuracy and interpretability is essential to promote broader acceptance and effective use of AI-integrated DT systems [105].

Hybrid modeling offers a promising approach by combining the strengths of physics-based and data-driven models. This architecture aims to preserve the interpretability and domain fidelity of physical models while leveraging the adaptability and predictive power of machine learning. In practice, however, many implementations treat these models as discrete components, for example, using physics-based simulations solely to generate training data for AI algorithms [122,123,124]. To unlock the full potential of hybrid modeling in DTs, deeper integration is needed. This includes embedding physical laws, constraints, and domain knowledge directly into AI inference processes, facilitating model generalization more reliably while remaining grounded in physical reality. Such approaches will not only improve model robustness but also foster greater operator confidence in AI-assisted decisions.

### 6.3. Standards and Benchmarks for Validation

Validation remains a critical yet challenging aspect of DT development, especially in AI-integrated systems. While the original DT concept proposed by Grieves and Vickers included a validation method based on complete interactive indistinguishability between the virtual and physical systems, this level of fidelity is not currently achievable with existing technology [6]. As a result, DT validation strategies have largely shifted toward case-specific approaches tailored to the application context and available modeling capabilities [98]. Like any emerging technology, the establishment of standardized benchmarks and validation protocols is essential, not only to ensure safety and sustainability but also to align DT development with industry performance and business objectives.

In AI-based DTs, validation becomes particularly complex due to the risks involved in testing predictive outputs in physical systems. For example, intentionally violating a RUL prediction to test its accuracy can result in costly failures or unsafe conditions. Instead, standard practice involves withholding a portion of the data from model training to use as a test set for evaluating prediction accuracy and generalization performance [125]. For process optimization applications, validation is typically performed within the DT environment through simulation, allowing new solutions to be assessed virtually before real-world implementation [7].

In scenarios where direct physical validation is not feasible, uncertainty quantification becomes a vital tool for increasing trust in AI-driven predictions [126]. Several case studies have implemented techniques such as confidence intervals to indicate prediction reliability and to explore the relationship between training data volume and model accuracy [123,127]. These practices can help mitigate the “black box” nature of many AI algorithms by transforming discrete outputs into transparent, probabilistic recommendations. Such transparency supports more informed decision-making and contributes to the broader acceptance of AI-integrated DT systems in safety-critical manufacturing applications.

### 6.4. Strategic Frontiers and Long-Term Outlook

Future research and development in AI-integrated DTs present a wide array of opportunities to further advance smart manufacturing. DTs are increasingly positioned as central hubs for synchronized operations, integrating seamlessly with enterprise-level systems such as Manufacturing Execution Systems (MES) and Enterprise Resource Planning (ERP) platforms [98]. For example, the DTSF paradigm proposed by Tao et al. [7] incorporates enterprise data to facilitate real-time production planning and adaptive process control, illustrating the growing role of DTs as operational decision-making engines across the factory floor [106].

Despite this progress, several strategic gaps remain. A lack of standardization [30,90,96,99,128] and semantic modeling frameworks [30,128] continues to hinder the scalability and interoperability of DT systems. Most notably, the issue of AI explainability remains a critical barrier to broader adoption. As DTs increasingly support autonomous decisions, transparency and interpretability of AI-driven outputs are essential for facilitating user trust and promoting accountability in high-stakes industrial environments [99]

As DT ecosystems become more complex and interconnected, supporting technologies are emerging to address key limitations. Blockchain, for instance, offers secure and transparent data exchange across distributed DT networks. Simultaneously, AR/VR interfaces are redefining how users engage with DTs by enabling immersive operator training, remote validation, and real-time system oversight [100,129]. These innovations enhance both functionality and accessibility, facilitating more intelligent and interactive manufacturing environments.

Realizing the full potential of next-generation DTs will require more than technical advancement; it will depend on strategic alignment across systems, people, and institutions. Cross-sector collaboration between academia and industry is essential to harmonize standards, promote knowledge transfer, and accelerate innovation. Equally important is the development of a digitally skilled workforce capable of operating, interpreting, and improving DT-enabled systems. The long-term success of DTs thus rests on an integrated approach that aligns technological innovation with workforce development and organizational transformation [69,70,77,83,98,129].

## 7. Conclusions

AI-integrated DTs are transforming the manufacturing landscape, serving as a cornerstone for the Fourth Industrial Revolution. By combining physical systems with the corresponding virtual models through real-time data exchange and intelligent algorithms, DTs transform reactive operations into proactive, self-optimizing processes. The evolution of DTs, particularly supported by DRL and advanced machine vision, allows them to expand their functionalities from mere simulation to knowledge acquisition, task scheduling, and action optimization, embodying the true essence of cognitive automation.

These technologies are gradually being adopted in diverse manufacturing applications from predictive maintenance and process optimization in CNC machining to autonomous robotics and large-scale industrial printing. Their value lies in their enhanced efficiency, cost-effectiveness, and improved product quality. The ability to train complex AI models safely and affordably within virtual twin environments significantly facilitates the development and deployment of manufacturing intelligence.

Despite technical challenges related to data fidelity, the virtual–physical realism gap, and the exploration–exploitation trade-off in AI training, AI-driven DTs will continue to advance toward increasingly sophisticated, adaptive, and resilient manufacturing systems. The integration of human expertise with robust data management strategies will further enhance their transformative potential, facilitating truly intelligent and adaptive industrial processes that are responsive to operational changes and unforeseen disruptions. The benefits of AI-driven DTs represent not merely a technological advancement, but a fundamental paradigm shift of manufacturing for the future.

## Figures and Tables

**Table 1:** Comparison of AI methodologies supporting digital twin applications in manufacturing.

AI Method	Representative DT-Enabled Use Cases in Manufacturing	References
Supervised Learning	Fault diagnosis; RUL estimation; product quality prediction; adaptive decision models using transfer learning; continual learning for concept drift	[5,17,28,29,30,31,32,33]
Unsupervised Learning	Anomaly detection in high-value manufacturing (e.g., semiconductor bonding); clustering operating states; edge-computing fault monitoring; CPC for predictive maintenance	[34,35,36,37,38]
DL (Discriminative)	Surface defect detection; tool wear monitoring; assembly quality prediction using Vision Transformers	[41,42,43,44]
DL (Generative AI)	Early-stage design exploration using LLMs; cognitive DT integration via ChatGPT with DES + IoT systems; design-for-manufacturability support through generative model–driven design alternatives	[45,46,47]
Reinforcement Learning	Process scheduling in rolling mills; robotic arm training in DT simulators; multi-stage S2R policy transfer pipelines	[50,51,52,53]
Hybrid/Physics-Informed AI	Physics-guided modeling for metal additive manufacturing; PDE-constrained PINN-based DT modeling; physics-informed prognostics under scarce data	[57,58,59,60]

## Data Availability

Data sharing is not applicable.
